# Tales not from the clinic!

**DOI:** 10.1177/20533691221121995

**Published:** 2022-08-23

**Authors:** Elham Amini, Paula Briggs

**Affiliations:** 1 Department of Sociology, Social Policy and Criminology, University of Liverpool, Liverpool, UK; 2 156761 Liverpool Women’s Hospital, Liverpool, UK

This inciteful tale explains why women from different ethnic and cultural backgrounds manage the menopause differently. There are some striking similarities to current menopause management in the UK including the reference to ovarian failure and the fear and negativity associated with that terminology, along with over medicalisation.

## Introduction

I need to start with a ‘warning’ to emphasise that this is not a tale from the clinic, as I do not work in the clinic. My background is midwifery and I used to work in different clinics and hospitals in Iran. I graduated as a midwife from Tehran University of Medical Sciences in Iran in 1996 and practised midwifery for more than seven years in different settings: a reproductive health research centre to which ‘infertile women’ had been referred, a public clinic and also in the labour ward of a private hospital in Tehran, called Kasra. Working in Iran, an Islamic country, my professional role involved caring for many different women, from different age groups and socio-economic backgrounds. I frequently heard about the most hidden and private parts of their lives. Most of the women who became menopausal thought of this as a kind of ‘dysfunction’ or ‘disease’ which was inevitable and a biological consequence of the ageing process; yet they felt guilty that they could not be a ‘good wife’ and ‘sexually satisfy their husbands’. They also carried within themselves a fear of losing their husbands due to ‘falling short in the fulfilment of their duties’, so they came to clinics in order to have ‘their problem fixed’. My feelings as their midwife were both of concern and of frustration, which led me to continue my studies, but in a critical social scientific direction, and I was awarded my PhD in Sociology of Health and Gender from Durham, UK. I did my research on gendered and sexual experiences of Iranian Muslim menopausal women and this paper is based on one section of that research.

I aim to encourage thinking about menopause and menopausal experiences as this relates to at least two key aspects of women’s lives, namely, gender and ethnicity. I address menopause and the menopause experience as a gendered, embodied and lived phenomenon in Iranian Muslim women. Menopause is characterised both by cultural constraint and by the agency of individuals which resides in the fact that participants negotiate and mediate gender power through their bodies, as well as in the specific ways that they interpret dominant cultural symbolism and meanings. So, instead of focussing on menopausal symptoms, such as hot flushes, anxiety and depression and vaginal dryness and how to fix them by ‘giving hormones’, I aim to explain why menopause serves as a negative shift in women’s social status and their self-appraisals. My other question is how a natural and inevitable part of a woman’s life course has been shaped as a catastrophe by celebrities and some journalists.

Therefore, menopause in this paper is not limited to the biological and hormonal aspects (although they are not ignored), but it is considered as a dynamic social interaction between, at the macro-level, socio-cultural structures and at the micro-level, personal, embodied responses to these normative structures. Menopause indeed has a material part, which is about the hormones and cessation of bleeding, but we live with our cultural bodies.^1,2^ We understand our physical body and the experiences related to it through meanings which are embedded in socio-cultural structures of our society.^3^ This is particularly relevant to the experience of gendered embodiment and the phenomena such as menstruation and menopause. Both medical discourse and cultural structure shape the meaning of menopause, influencing women’s lived experiences.

I stress that there is a need to understand the menopausal experiences of women from Global South and ethnic minorities in Global North. The underlying assumption that considers embodied gendered experience of menopause to be similar around the world and conceptualise it based on the Western white women’s experiences not only ignores the different experience of women from Global South or ethnic minorities in Global North, but also shapes epistemic exclusion within the field of knowledge. This makes a kind of knowledge that exacerbates social inequality and marginalisation. It also reveals the power game in knowledge and hierarchical relationship which makes otherness.

This paper will begin by providing an overview on the research and its methodology. Then, I will address how Iranian women in this research understood their menopausal experiences and at the end explain their sexual experiences during menopause.

## Method and the context

As noted previously, this paper is a part of a broader project regarding older women’s health and experience of menopause in Iran. In winter 2014 and spring 2015, I conducted 30 individual biographical interviews with women who regularly attended religious classes in Tehran and Karaj. 99.5% of the Iranian population is Muslim^4^ but not all are actively practicing Islam. Being religious is one of the essential characteristics of my target group, therefore it was important to find women who practiced Islam. Thus, I recruited the research participants from the women who regularly attended religious or Quran classes in Tehran and Karaj. To access different socio-economic classes, I chose five Quran classes from different geographical areas of Tehran: North, Northeast, North Centre, Southeast and ‘Downtown’. Additionally, one Quran class was located in Karaj, the fourth largest city of Iran and 20 kilometres west of Tehran. People who cannot afford to live in Tehran often relocate to Karaj.

The median age of menopause in Iran is reported as 49.9 in urban, 49.2 in rural areas and 49.6 years in the total population.^5^ Therefore, although menopause is perceived as a symbol of ‘old age’,^6–8^ women who become menopausal are typically younger than 50 and are actually middle, rather than old-aged.

### Understanding menopause and menopausal experiences

There was a hegemony of silence on menopause and menopausal experiences in the women interviewed. Most of the women in this research claimed that I was the first person they had ever talked to in depth about ‘these issues’ and they felt ‘peaceful’ after their interviews. However, interviews were full of emotions (crying, sobbing, shaking, picking at cuticles,…). Most of the women spoke in a faint voice when they wanted to disclose their menopausal experiences or referred to themselves as a ‘menopausal woman’ which shows menopause is a taboo subject.

At menopause, a bodily change (cessation of menstruation) causes the subjective experiences which are affected by the socio-cultural structure (gender order, medicalisation and shared meanings) that shapes understanding of the experience. The language around menopause in Iran is a good example of this. In the Farsi language, menopause is called یائسگی (Yaesegi) from the root یأس (Yaas), which means disappointment. Accordingly, menopause, یائسگی, in Farsi, means a time of despair and disappointment.

All the women who participated in this research believed that the menopause marked a pivotal point in their identity. A woman, before the menopause, is considered to be ‘a true woman’, whereas a menopausal woman can no longer perform the role of child-bearer. Thus, menopause can be a symbol of sterility and has symbolic meaning for the end, or a transition to a different kind, of femininity.^6,7,9.^ Menstruation is a symbol of fertility with cessation of menstruation representative of failure of fertility. The physical loss of menstrual periods at menopause plays a crucial role in the identity of menopause highlighting centrality of menstruation in relation to menopause. It emerged, from the women’s accounts, that the passage from womanhood to menopause is measured as a bodily event, when the normal menstrual cycle undergoes a variety of changes: irregularity of menstruation and for some women, other physical experiences, such as hot flushes and vaginal dryness.

Most of the women in this study became aware of their menopause through diagnosis by medical staff while others found out about it through the knowledge that they had obtained from their peers, before their menopause had started.

Eftekhar, a 54-year-old, a teacher with Masters in Islamic theology explained that she did not like her menopause and reported that when her irregular menses started, she went to see a gynaecologist:

‘*Yes, I don’t like it. I became menopausal very early; I think I was 47 or 48. You know, I did the blood exam to find out if I had become menopausal or not. When the result was ready, I went to see my gynaecologist. I will never forget that day, when she saw my exam; she looked at me as if I had cancer and was going to die! At first, I was really terrified. Then she told me she was sorry, and I was too young to become menopausal, but my exam showed that I had the menopause’.*

Eftekhar’s feelings towards her menopause (dislike), is constructed by the way that she was diagnosed as a menopausal woman by medical staff. Also, the gynaecologist’s attitude towards the menopause is based on the medical texts which refer to menopause as a ‘failure’, and can refer to ovarian failure, reproductive failure or hormonal failure.^10,11^ After examining many international medical textbooks about menopause, Nilhand and Lyons^12^ highlight that medical knowledge still dominantly considers menopause as a failure and a precursor to disease.

Another example, which shows how biological age and medical definitions frame and structure meanings and understanding of women’s experiences in relation to menopause is that of Hoda, a 66-year-old housewife, who reached the menopause when she was 54 years old. She was tested by her gynaecologist when she was ‘*around 52’* to examine her hormone levels, to find out whether she was in the perimenopause. Although she did not have any symptoms or signs of menopause at that time, the doctor merely did the test due to her biological age and through the test result, Hoda realised that she was in the perimenopause. In addition, despite the fact that she did not have any symptoms or signs, the doctor recommended that she start hormone replacement therapy, based merely on her biological age. She refused. These examples display the influence of biological age in relation to the medical authorities understanding of menopause and this defines women’s experiences.

Anis, a 50-year-old teacher with B.A in elementary education. She stated ‘*menopause is a monster, and it is the reason of [my] depression*’*:*

‘At the beginning, I thought, Oh! It would be good not to have menstruation’, but after a while my bone pain started, and then I became depressed. After my menopause nothing can make me happy. It was like a monster that captured my entire body. For example, now everyone is shopping for New Year, and they are happy, but for me it is something vain and meaningless’.

Interpreting cessation of menstruation as a gendered embodied phenomenon has had a pivotal role in shaping menopause as an illness. Amini and McCormack explained that,^6^ some Iranian women viewed menstruation as the excretion of ‘polluted’ blood and menopause was seen as a source of illness because it retained pollution^13^ in the body.

Samin, a 54-year-old housewife who became menopausal when she was 46 years old, said,

‘*When you have your menstruation, your dirty blood comes out of your body every month, but when this ceases, the dirty blood remains in your body. Therefore, menopause can’t be good and healthy, as it’s the source of all pains that we have, such as this terrible bone pain….’*

In Samin’s narration, menstrual blood plays an important role in her understanding of her menopause. Menstrual blood has been considered as dirty blood which can pollute the body and cause illness. In this regard, menopause is the time that dirty blood accumulates in the body and causes illness.

### Sexual experiences at menopause

Despite menopause being seen as a medical problem and a great loss, many participants used it as a mechanism to discuss their dissatisfaction with their sexual relationships. In the Iranian context that Civil Procedure law, part 1105, compels wives to fulfil the sexual needs of their husbands,^6,7^ menopause gives women a space to challenge the harmful sexual practices they encounter, particularly through using menopause as an excuse for rejecting unwanted sex. Most of the women cited their menopause as a reason to start a conversation with their husbands about sex, their attitude toward it and to negotiate their sexual desires within their marriage. For example, Zeinab, a 62-year-old woman who became menopausal when she was 50 years old, stated that her lack of sexual desire during menopause was ‘*not in her hands’* adding

‘*From the time when I started the menopause, I can say no to my husband [smiling], and he's going to get less upset over it, because it's not in my hands, it's because of my menopause*’. Thus, menopause enables a discussion about sex which had not occurred before, including a rejection of a women’s role based on the gender order. The menopausal body is considered ‘old’ in Iranian culture,^14^ and women used this to reject sexual intercourse, which they had previously endured as an obligation or necessity.

Along with the gendered structure of Iran, medical discourse by introducing ‘natural’ sex is the other power that has a significant effect on menopausal women’s sexual lives. In this study, all the women who had the experience of a gynaecologist’s services during their menopause explained that it had been suggested to them that they use some medication such as oestrogen cream, lubricant or gel and even hormonal replacement therapy, to improve their sexual experiences. Some of the women who participated in this study were encouraged by a gynaecologist to undergo perineorrhaphy/perineoplasty cosmetic surgery, in order to enhance their sexual experiences or to prevent ‘problems’. These recommendations by medical experts demonstrate firstly, that the socio-cultural structure to which the medics respond, ignores sexual variation. The focus of medical experts is phallocentric vaginal sexual intercourse to the exclusion of other sexual practices. Secondly, by defining a ‘standard’ for sexual functionality, profoundly affects menopausal women’s sexual experiences.^15,16,17^ Thirdly, they shape a set of expectations for the type and quality of the menopausal women’s sexual activity, by presenting a ‘standard’ criterion, and then providing their interventions to reach these ‘standards’. Finally, the use of surgical and medical interventions suggests that this ‘standard’ is based on youthfulness with treatment rejuvenating the vagina in order to have ‘ideal’ sex. For example, when I asked Farideh, a 57-year-old teacher who became menopausal when she was 49 years old about her sexual activity after her menopause, she said,

‘After my menopause, it became painful. The gynaecologist told me it’s because of menopause and I have to use some gels or hormonal creams or start hormonal therapy. I did hormonal therapy for a while, but now just use those gels and creams’.

And, she continued by explaining the type of sex that she enjoyed:

‘I like the hugging and these things, but not the other part. But, you know, I’m his wife and it’s my responsibility to do it,…’

Habibeh, a 69-year-old, retired nursery teaching assistance who became menopausal when she was 50 was the only participant who had had the perineorrhaphy surgery, she said,

‘It was for a while, that my husband repeatedly told me he was not satisfied, I mean, pardons me, he said it had been very loose. I went and saw a gynaecologist. She told me it usually happened after menopause and especially after normal delivery; you know I had three normal deliveries, so I think it was normal that it happened to me. She told me the only solution was undergoing surgery to tighten it, and she said after doing the surgery it would be like the time, I was young (smiling) and it would help my husband’s satisfaction. It wasn’t a very difficult surgery, so I did it. But to tell the truth, now it’s more painful for me...My husband still nags sometimes. You know, maybe because I don’t like it. I like the cuddling and touching of it, but I have the fear of losing my husband if I don't do it’.

Mahdieh, a 51-year-old teacher who became menopausal when she was 50 years old also mentioned,

‘I like the times that he sat near me and holding my hands or hugging me, you know, I think for most of the women the sex act is not important. But I have to pretend to have the same desire for the other one as well, as I want to keep my marriage safe’.

Medical discourse along with the patriarchal structure in Iran prioritises men’s sexual needs over their wives’. Menopausal women’s sexual relationship links to broader socio-cultural conditions. Subsequently, we cannot consider medication such as oestrogen cream or hormone replacement therapy as the only solution for menopausal women’s sexual ‘problems’. Although the women in this research desired the other types of intimacy such as hugging and kissing, they did not call it sex as they believed it is not ‘real sex’*.* Medical experts by defining phallocentric sex as the only ‘real sex’ maintain a normative definition of sex and sexual relationships which promotes gendered hierarchies.

## Conclusion

In this study, Iranian women’s understanding of their menopause and menopausal experiences was examined. The focus on Iranian’s women experiences highlighted the significant role of socio-cultural context which makes menopause a taboo subject and shapes hegemony of silence on menopausal experiences. Linking biology to culture and the socio-cultural structure of the society, demonstrated that the patriarchal structure in Iran along with medical discourse frames the menopause as hormonal failure and deficiency, and thus as a purely biological issue. Subsequently, women understood their menopausal experiences as an illness and a social stigma which makes it difficult for women to talk about it.

I want to finish this paper by telling my last tale which is about the celebrities and their approach to menopause. Last week, I received an email from the ‘menopause support group’ at my workplace. The ‘Menopause Bus’ would be at the Albert Dock on Wednesday 20th July and the producer is looking for ‘*case studies of women of ethnic minorities who have experienced difficulties surrounding myths and stereotypes of the menopause for them’*. Although they mentioned the stereotypes of the menopause, the producer by ignoring the cultural taboo aspect of menopause, assumes it is easy for the ethnic minorities to speak up about their menopause in a crowded public space such as the Albert Dock and in front of the camera. This, alongside my findings reiterates the importance of acknowledging culture and gender order in creating women’s menopausal experiences and its implications in shaping their health behaviours and reproductive health.
